# Host associations of mosquitoes at eastern equine encephalitis virus foci in Connecticut, USA

**DOI:** 10.1186/s13071-016-1765-1

**Published:** 2016-08-30

**Authors:** John J. Shepard, Theodore G. Andreadis, Michael C. Thomas, Goudarz Molaei

**Affiliations:** 1 Department of Environmental Sciences, and Center for Vector Biology & Zoonotic Diseases, The Connecticut Agricultural Experiment Station, 123 Huntington Street, New Haven, CT 06511 USA; 2 Department of Epidemiology of Microbial Diseases, Yale School of Public Health, 60 College Street, P.O. Box 208034, New Haven, CT 06520-8034 USA

**Keywords:** Mosquito blood-feeding, Eastern equine encephalitis virus foci, Epidemic/epizootic transmission, “Bridge vector”, Connecticut

## Abstract

**Background:**

Eastern equine encephalitis virus (EEEV) is a highly pathogenic mosquito-borne arbovirus, with active transmission foci in freshwater hardwood swamps in eastern North America, where enzootic transmission is maintained between the ornithophilic mosquito, *Culiseta melanura,* and wild passerine birds. The role of other locally abundant mosquito species in virus transmission and their associations with vertebrate hosts as sources of blood meals within these foci are largely unknown but are of importance in clarifying the dynamics of enzootic and epidemic/epizootic transmission.

**Methods:**

Blood-engorged mosquitoes were collected from resting boxes at four established EEEV foci in Connecticut during 2010–2011. Mosquitoes were identified to species, and the identity of vertebrate hosts was determined based on mitochondrial cytochrome *b* and/or cytochrome *c* oxidase subunit I gene sequences of polymerase chain reaction products.

**Results:**

The vertebrate hosts of 378 (50.3 % of engorged mosquitoes) specimens, representing 12 mosquito species, were identified. *Culiseta morsitans* (*n* = 54; 67.5 %), *Culex restuans* (*n* = 4; 66.7 %), and *Cx. pipiens* (*n* = 2; 100 %) acquired blood meals exclusively from avian hosts, whereas *Aedes cinereus* (*n* = 6; 66.7 %), *Ae. canadensis* (*n* = 2; 100 %), and *Ae. stimulans* (*n* = 1; 100 %) obtained blood meals solely from mammals. Species that fed opportunistically on both avian and mammalian hosts included: *Ae. thibaulti* (*n* = 21 avian, and *n* = 181 mammalian; 57.2 %), *Anopheles punctipennis* (*n* = 8 and *n* = 40; 44.0 %), *An. quadrimaculatus* (*n* = 1 and *n* = 23; 35.7 %), *Coquillettidia perturbans* (*n* = 3 and *n* = 3; 46.2 %) and *Ae. abserratus* (*n* = 1 and *n* = 2; 23.1 %). *Culex territans* obtained blood meals from avian and amphibian hosts (*n* = 18 and *n* = 5; 26.6 %). Mixed blood meals originating from both avian and mammalian hosts were identified in *An. quadrimaculatus* (*n* = 1), and *Cx. territans* (*n* = 2).

**Conclusions:**

Our findings indicate that wood thrush, tufted titmouse, and a few other avian species serve as hosts for mosquitoes, and likely contribute to amplification of EEEV. Our study supports the role of *Cs. morsitans* in enzootic transmission of EEEV among avian species. *Culex territans* will seek blood from multiple vertebrate classes, suggesting that this species may contribute to epizootic transmission of the virus. Our findings support roles for *Cq. perturbans* and *An. quadrimaculatus* as epidemic/epizootic vectors to humans, horses, and white-tailed deer. Despite its abundance, the potential of *Ae. thibaulti* to serve as a “bridge vector” for EEEV remains unclear in the absence of any definitive knowledge on its competency for the virus. The contribution of white-tailed deer to the dynamics of EEEV transmission is not fully understood, but findings indicate repeated exposure due to frequent feeding by vector competent mosquito species.

## Background

Eastern equine encephalitis virus (EEEV) (*Togaviridae*: *Alphavirus*) is a highly pathogenic mosquito-borne arbovirus that is capable of causing severe neurological disease and fatalities in humans and equines [1–3]. Active transmission foci are largely confined to freshwater hardwood swamps throughout the eastern half of North America extending from the Gulf of Mexico to southern Canada and the upper Midwest. In the northeastern United States, enzootic transmission of EEEV occurs seasonally from mid-May through early October, where the virus is maintained between the ornithophilic mosquito, *Culiseta melanura* (Coquillett), and wild passerine birds. EEEV activity has been historically episodic; however, more recently the frequency of virus activity has changed, and human and equine disease incidence has increased in the northeastern United States (e.g. in Connecticut, Maine and Vermont) [3–9].

**Fig. 1:**
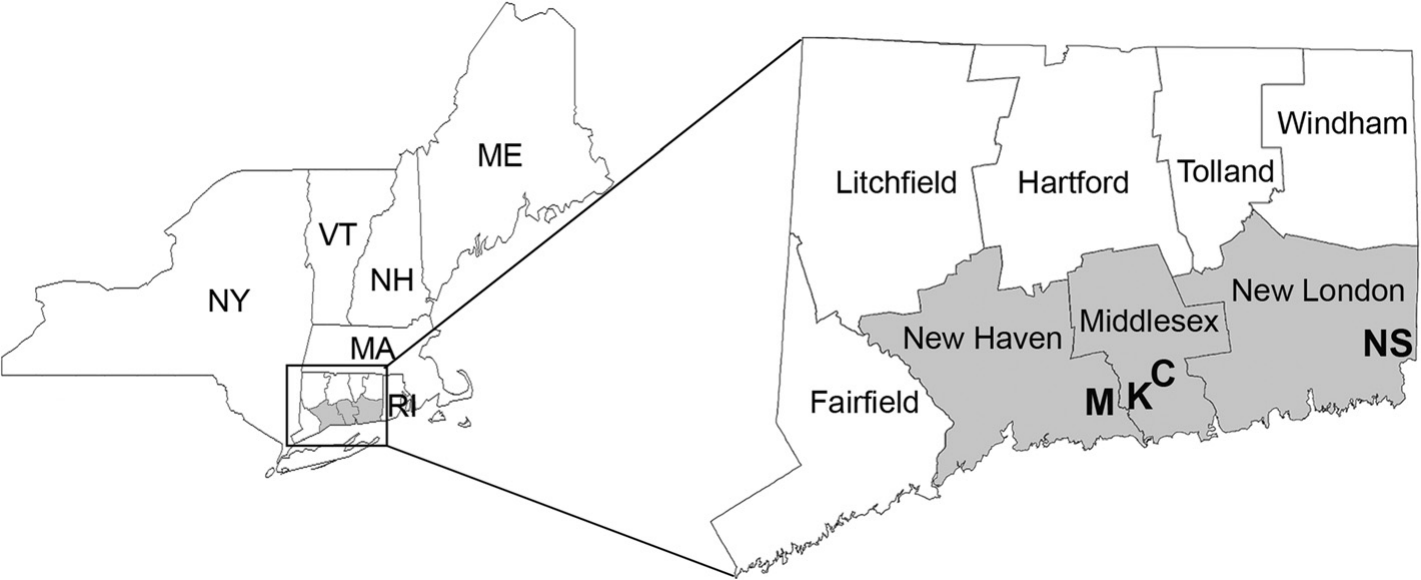
Location of four eastern equine encephalitis virus foci study sites in Connecticut, 2010–2011. *Abbreviations*: C, Chester; K, Killingworth; M, Madison; NS, North Stonington

Although several studies have reported on the role of *Cs. melanura* in transmission of EEEV among wild birds [10–16], comparatively little is known about the contribution of other mosquito species to enzootic and/or epidemic/epizootic transmission to mammalian hosts including humans and equines. A number of mosquito species, including *Aedes canadensis* (Theobald), *Aedes sollicitans* (Walker), *Aedes vexans* (Meigen), *Coquilletidia perturbans* (Walker), *Culex salinarius* Coquillett, *Anopheles punctipennis* (Say), and *Anopheles quadrimaculatus* Say, have been considered as potential “bridge vectors” of EEEV across its geographic range, based on virus isolations from field-collected mosquitoes, vector competence evaluations, and host association studies [17–22]; however, the details of their contribution in various virus foci has not been fully realized.

Recently, a study on the role of *Cs. melanura* and several avian hosts in transmission and amplification of EEEV was carried out in order to better understand the dynamics of virus transmission at four enzootic foci in Connecticut [16]. The present investigation is an extension of the latter study. Our objective was to examine the host utilization of other locally abundant mosquito species in an attempt to assess their potential contribution to EEEV transmission in these locales. Accordingly, mosquitoes were collected from the virus foci, and vertebrate hosts were identified by analysis of mitochondrial cytochrome *b* and/or cytochrome *c* oxidase subunit I gene sequences.

## Methods

### Study sites

Mosquitoes were collected from four historic EEEV foci in Chester (41°23.233′N, 72°29.564′W), Killingworth (41°20.217′N, 72°34.322′W), Madison (41°21.628′N, 72°39.131′W), and North Stonington (41°26.175′N, 71°49.845′W), Connecticut during 2010–2011 (Fig. 1). These freshwater swamps were chosen on the basis of prior isolations of EEEV from mosquito pools as previously described [16]. Common canopy tree species included red maple, *Acer rubrum* Linnaeus, and Atlantic white cedar, *Chamaecyparis thyoides* (L.), which support underground crypt habitats formed by tree root mats and windthrow pools formed by uprooted trees, where *Cs. melanura* larvae were commonly encountered. Well-developed understories were comprised of mountain laurel, *Kalmia latifolia* L., spicebush, *Lindera benzoin* (L.), *Sphagnum* spp. and ferns.

### Mosquito sampling

Mosquitoes were collected from 120 resting boxes and/or stackable fiber pots placed adjacent to the red maple/Atlantic white cedar swamps according to previously described methods [16, 23, 24]. The number of resting boxes per study site was variable, and included Chester and Madison (*n* = 45 each), and Killingworth and North Stonington (*n* = 15 each). Resting boxes were examined daily from May to October 2010 and June to October 2011. Mosquitoes were removed from the resting boxes using hand-held mechanical aspirators, transferred to coolers containing dry ice, and transported to the laboratory. Identification of female mosquitoes to species was carried out on dry ice with the aid of a dissecting microscope using morphological keys [25, 26]. Individual mosquitoes with visible evidence of blood meals were placed in 1.5 ml microcentrifuge tubes, labeled with a unique number, and stored in an ultra-low temperature freezer.

### DNA isolation and blood meal identification

Blood-engorged abdomens were removed using disposable single-edge razor blades with the aid of a dissecting microscope. DNA was extracted from individual abdominal contents using DNAzol BD (Molecular Research Center, Cincinnati, OH, USA) according to the manufacturer’s recommendations with modifications as previously described [12, 27, 28]. Partial mitochondrial cytochrome *b* gene sequences were obtained through screening extracted DNA by polymerase chain reaction (PCR), using avian- and mammalian-specific primers. Avian-specific primers, 5′-GAC TGT GAC AAA ATC CCN TTC CA-3′ (forward) and 5′-GGT CTT CAT CTY HGG YTT ACA AGA C-3′ (reverse), yielded a 508 bp fragment. Mammalian-specific primers, 5′-CGA AGC TTG ATA TGA AAA ACC ATC GTT G-3′ (forward) and 5′-TGT AGT TRT CWG GGT CHC CTA-3′ (reverse), produced a 772 bp fragment. A Taq PCR Core kit (Qiagen, Valencia, CA, USA) was used for PCR reactions according to the manufacturer’s instructions in a Veriti thermal cycler (Applied Biosystems, Foster City, CA, USA). Thermal cycling conditions and reactions were as previously described [12, 27, 28].

Additional primer sets utilized to screen *Cx. territans* for evidence of blood feeding on amphibian and reptilian hosts included: (i) a primer set with a broad target group based on mitochondrial cytochrome *c* oxidase subunit I (COI) gene [29]; (ii) an amphibian-primer set based on mitochondrial cytochrome *b* gene [30]; and (iii) a reptilian-specific primer set targeting mitochondrial cytochrome *b* gene [30]. The COI primer set, 5′-TGT AAA ACG ACG GCC AGT TCT CAA CCA ACC ACA ARG AYA TYG G-3′, (forward) and 5′-CAG GAA ACA GCT ATG ACT AGA CTT CTG GGT GGC CRA ARA AYC A-3′ (reverse), targeted a 648 bp region. Thermal cycling conditions for COI included an initial denaturation for 2 min at 94 °C, followed by 40 cycles of denaturation at 94 °C for 30 s, annealing at 45 °C for 45 s, and primer extension at 72 °C for 1 min. The final cycle was completed with 10 min of extension at 72 °C.

The amphibian-specific primer set, 5′-THC TNT CNG CHG CCC CVT A-3′ (forward) and 5′-GAG CGD AGR ATN GCR TAR GC-3′ (reverse), targeted a 402 bp region. Thermal cycling conditions for the amphibian-specific primer set included an initial denaturation for 10 min at 95 °C, followed by 36 cycles of denaturation at 94 °C for 30 s, annealing at 54 °C for 45 s, and primer extension at 72 °C for 90 s. The final cycle was completed with 7 min of extension at 72 °C.

The reptilian-specific primer set, 5′-GGN TCR TCC AAC CCA AYW G-3′ (forward) and 5′-TTT DGC DAD DGG DCG RAA N-3′ (reverse), targeted a 518 bp region. Thermal cycling conditions for the reptilian-specific primer set included an initial denaturation for 10 min at 95 °C, followed by 36 cycles of denaturation at 94 °C for 30 s, annealing at 50 °C for 40 s, and primer extension at 72 °C for 1 min. The final cycle was completed with 7 min of extension at 72 °C.

Sequencing reactions were carried out on a 3730xL DNA Analyzer (Applied Biosystems) at the Keck Sequencing Facility, Yale University, New Haven, Connecticut. Nucleotide sequences were analyzed and annotated using ChromasPro version 1.7.5 (Technelysium Pty, Ltd., Tewantin, Australia), and the identity of host species were determined by comparisons to the GenBank sequence database (using the BLAST search) maintained at the National Center of Biological Information [31].

## Results

In our earlier investigation, a total of 1798 *Cs. melanura* with visible blood meals were collected from the four virus foci, and blood meal sources were identified in 1127 (62.7 %) specimens by DNA sequencing [16]. In the present study, a total of 2773 engorged and unengorged female mosquitoes, representing 18 species (excluding *Cs. melanura*), were collected from 120 resting boxes at four virus foci during 2010–2011. The blood meal sources of 378 (50.3 % of engorged mosquitoes) specimens, comprising 12 species, were identified to vertebrate species (Table 1). Infrequently collected mosquito species with no evidence of blood meal or inconclusive blood meal results included: *Aedes excrucians* (Walker), *Aedes trivittatus* (Coquillett), *Anopheles barberi* Coquillett, *Anopheles crucians* Wiedemann, *Psorophora ferox* (von Humboldt) and *Uranotaenia sapphirina* (Osten Sacken).

**Table 1:** Number and percentage of avian-, mammalian- and amphibian-derived blood meals identified from 12 mosquito species in four eastern equine encephalitis virus foci in Connecticut, 2010–2011

Mosquito species	Avian No. (%)	Mammalian No. (%)	Amphibian No. (%)	Mixed No. (%)	Blood meal ID^a^ No. (%)	Engorged specimens
*Aedes thibaulti*	21 (10.4)	181 (89.6)			202 (57.2)	353
*Culiseta morsitans*	54 (100)				54 (67.5)	80
*Anopheles punctipennis*	8 (16.7)	40 (83.3)			48 (44.0)	109
*Anopheles quadrimaculatus*	1 (4.0)	23 (92.0)		1 (4.0)	25 (35.7)	70
*Culex territans*	18 (72.0)		5 (20.0)	2 (8.0)	25 (26.6)	94
*Aedes cinereus*		6 (100)			6 (66.7)	9
*Coquillettidia perturbans*	3 (50.0)	3 (50.0)			6 (46.2)	13
*Culex restuans*	4 (100)				4 (66.7)	6
*Aedes abserratus*	1 (33.3)	2 (66.7)			3 (23.1)	13
*Culex pipiens*	2 (100)				2 (100)	2
*Aedes canadensis*		2 (100)			2 (100)	2
*Aedes stimulans*		1 (100)			1 (100)	1
Total	112	258	5	3	378	752

^a^Blood meal ID, number of blood meals successfully identified to species level

*Culiseta morsitans* (Theobald) (*n* = 54; 67.5 % of engorged mosquitoes), *Culex restuans* Theobald (*n* = 4; 66.7 %) and *Culex pipiens* L. (*n* = 2; 100 %) acquired blood meals exclusively from avian hosts; whereas *Aedes cinereus* Meigen (*n* = 6; 66.7 %), *Ae. canadensis* (*n* = 2; 100 %), and *Aedes stimulans* (Walker) (*n* = 1; 100 %) obtained blood meals solely from mammals (Table 1). Mosquito species that opportunistically obtained blood meals from avian and mammalian hosts included: *Aedes thibaulti* Dyar & Knab (*n* = 21 avian and *n* = 181 mammalian; 57.2 %), *An. punctipennis* (*n* = 8 and *n* = 40; 44.0 %), *An. quadrimaculatus* (*n* = 1 and *n* = 23; 35.7 %), *Cq. perturbans* (*n* = 3 and *n* = 3; 46.2 %), and *Aedes abserratus* (Felt & Young) (*n* = 1 and *n* = 2; 23.1 %) (Table 1). *Culex territans* Walker obtained blood meals from avian and amphibian hosts (*n* = 18 and *n* = 5; 26.6 %). Mixed blood meals originating from both avian and mammalian hosts were identified from specimens of *An. quadrimaculatus* (*n* = 1) and *Cx. territans* (*n* = 2) (Table 1).

Results of 112 avian-derived blood meals identified from nine mosquito species are presented in Table 2. Twenty-four bird species, representing 14 families in six orders, were identified as hosts. Passeriformes constituted 92.0 % of the total avian-derived blood meals. The most frequently fed upon passerine families were Turdidae (Thrushes, 43.7 %), Paridae (Chickadees and Titmice, 25.2 %), Icteridae (Blackbirds, 12.6 %), Viroenidae (Vireos, 8.7 %) and Mimidae (Mockingbirds and Thrashers, 3.9 %). Other avian orders included: Accipitriformes (4.5 %), and Anseriformes, Columbiformes, Cuculiformes and Piciformes (0.9 % each). The wood thrush *Hylocichla mustelina* (Gmelin) was the most frequent avian host (*n* = 39; 34.8 %), followed by the tufted titmouse, *Baeolophus bicolor* L. (*n* = 21; 18.8 %), red-winged blackbird, *Agelaius phoeniceus* (L.) (*n* = 7; 6.3 %), American robin, *Turdus migratorius* L. and yellow-throated vireo, *Vireo flavifrons* Vieillot (*n* = 6; 5.4 % each). Other avian hosts included: black-capped chickadee, *Poecile atricapillus* L., common grackle, *Quiscalus quiscula* (L.) (*n* = 5; 4.5 % each) and gray catbird, *Dumetella carolinensis* (L.) (*n* = 4; 3.6 %). Sixteen additional bird species infrequently served as hosts (Table 2).

**Table 2:** Number and percentage of avian-derived blood meals identified from nine mosquito species in four eastern equine encephalitis virus foci in Connecticut, 2010–2011

Order	Family	Species	RC	*Ae. abserratus*	*Ae. thibaulti*	*An. punctipennis*	*An. quadrimaculatus*	*Cq. perturbans*	*Cs. morsitans*	*Cx. pipiens*	*Cx. restuans*	*Cx. territans*	Total
				No. (%)	No. (%)	No. (%)	No. (%)	No. (%)	No. (%)	No. (%)	No. (%)	No. (%)	
Pass.	Turdidae	Wood thrush	S			1 (12.5)			34 (63.0)	1 (50.0)	1 (25.0)	2 (11.1)	39
	Turdidae	American robin	P, T	1 (100)	1 (4.8)			1 (33.3)	3 (5.6)				6
	Paridae	Tufted titmouse	P		8 (38.1)	2 (25.0)		1 (33.3)	3 (5.6)	1 (50.0)		6 (33.3)	21
	Paridae	Black-capped chickadee	P		3 (14.3)	1 (12.5)		1 (33.3)					5
	Icteridae	Red-winged blackbird	P, T		4 (19.0)	2 (25.0)			1 (1.9)				7
	Icteridae	Common grackle	P, T		2 (9.5)				1 (1.9)			2 (11.1)	5
	Icteridae	Brown-headed cowbird	P, T			1 (12.5)							1
	Vireonidae	Yellow-throated vireo	S		2 (9.5)							4 (22.2)	6
	Vireonidae	Warbling vireo	S									2 (11.1)	2
	Vireonidae	Red-eyed vireo	S								1 (25.0)		1
	Mimidae	Gray catbird	S						3 (5.6)		1 (25.0)		4
	Polioptilidae	Blue-gray gnatcatcher	S				1 (100)						1
	Emberizidae	Chipping sparrow	S									1 (5.6)	1
	Emberizidae	Field sparrow	S		1 (4.8)								1
	Sturnidae	European starling	P						1 (1.9)				1
	Cardinalidae	Northern cardinal	P						1 (1.9)				1
	Cardinalidae	Scarlet tanager	S						1 (1.9)				1
Acci.	Accipitridae	Broad-winged hawk	S						2 (3.7)				2
	Accipitridae	Red-tailed hawk	P						1 (1.9)			1 (5.6)	2
	Accipitridae	Sharp-shinned hawk	P						1 (1.9)				1
Anser.	Anatidae	Canada goose	P								1 (25.0)		1
Columb.	Columbidae	Mourning dove	P						1 (1.9)				1
Cucul.	Cuculidae	Yellow-billed cuckoo	S			1 (12.5)							1
Pici.	Picidae	Northern flicker	P						1 (1.9)				1
		Total		1	21	8	1	3	54	2	4	18	112

*Abbreviations*: *Pass*. Passeriformes, *Acci*. Accipitriformes, *Anser*. Anseriformes, *Columb*. Columbiformes, *Cucul*. Cuculiformes, *Pici*. Piciformes, *RC* residency codes, *P* permanent resident (found year round in the state), *S* summer resident (present in the state during the nesting season), *T* transient

Results of the 258 mammalian-derived blood meals are shown in Table 3. Eight species were identified as the source of blood meals, but the majority (*n* = 230; 89.1 %) of feedings were from white-tailed deer, *Odocoileus virginianus* (Zimmermann). Human-derived blood meals were infrequent and only identified from *Ae. thibaulti*, representing 0.4 % (*n* = 1) of all mammalian-derived blood meals. Among the other seven mammalian species that served as hosts, the horse, *Equus caballus* L., was the most frequently fed upon (*n* = 19; 7.4 %), and identified in three mosquito species: *Ae. thibaulti* (*n* = 12), *An. quadrimaculatus* (*n* = 6) and *An. punctipennis* (*n* = 1). Infrequently fed upon mammalian hosts included: eastern chipmunk, *Tamias striatus* (L.) and sheep, *Ovis aries* L. (*n* = 2; 0.8 % each); and domestic cat, *Felis catus* L., eastern cottontail rabbit, *Sylvilagus floridanus* (Allen), raccoon, *Procyon lotor* (L.) and Virginia opossum, *Didelphis virginiana* Kerr (*n* = 1; 0.4 % each) (Table 3).

**Table 3:** Number and percentage of mammalian-derived blood meals identified from eight mosquito species in four eastern equine encephalitis virus foci in Connecticut, 2010–2011

Species	*Ae. abserratus*	*Ae. canadensis*	*Ae. cinereus*	*Ae. stimulans*	*Ae. thibaulti*	*An. punctipennis*	*An. quadrimaculatus*	*Cq. perturbans*	Total
	No. (%)	No. (%)	No. (%)	No. (%)	No. (%)	No. (%)	No. (%)	No. (%)	
White-tailed deer (*Odocoileus virginianus*)	2 (100)	2 (100)	4 (66.7)	1 (100)	163 (90.1)	38 (95.0)	17 (73.9)	3 (100)	230
Horse (*Equus caballus*)					12 (6.6)	1 (2.5)	6 (26.1)		19
Eastern chipmunk (*Tamias striatus*)			2 (33.3)						2
Sheep (*Ovis aries*)					1 (0.6)	1 (2.5)			2
Domestic cat (*Felis catus*)					1 (0.6)				1
Eastern cottontail rabbit (*Sylvilagus floridanus*)					1 (0.6)				1
Human (*Homo sapiens*)					1 (0.6)				1
Raccoon (*Procyon lotor*)					1 (0.6)				1
Virginia opossum (*Didelphis virginiana*)					1 (0.6)				1
Total	2	2	6	1	181	40	23	3	258

The mammalian host species in all mixed blood meals was white-tailed deer, two of which mixed with the warbling vireo, *Vireo gilvus* (Vieillot), and one with the chipping sparrow, *Spizella passerina* (Bechstein).

Amphibian species identified as hosts for *Cx. territans* included the green frog *Lithobates clamitans* (L.) (*n* = 3), wood frog, *L. sylvaticus* (LeConte) (*n* = 1) and gray tree frog, *Hyla versicolor* LeConte (*n* = 1).

## Discussion

Results obtained in this study provide additional knowledge on the host associations of 12 locally abundant mosquito species that inhabit four freshwater EEEV foci, and provide further insight on their potential roles in enzootic transmission among wild birds and epidemic/epizootic transmission of the virus to humans and equines in the region.

Overall, approximately half (50.3 %) of all mosquito blood meals were successfully identified to species level. This varied by species, however, ranging from fairly low success (26.6 % of 94 in the case of *Cx. territans*) to moderate success (67.5 % of 80 for *Cs. morsitans*). Therefore, these results should be interpreted with varying levels of caution, as the preponderance of unidentified meals could have originated from any variety of hosts. It is noteworthy that all mosquitoes with fresh or visible blood remnants were examined and positive identification and host species assignment were made when exact or nearly exact match were obtained. Sequences that did not meet the criteria were assumed unknown. Several factors contribute to successful identification of the blood meal source including the amount of vertebrate blood acquired by mosquitoes, digestion of the blood meal in the mosquito gut, rapid degradation of host DNA, the time between capturing mosquitoes and processing for blood meal analysis, quality and quantity of isolated DNA, quality of the sequences and availability of the species-specific target gene sequences in the GenBank database, the degrees of sequence homology among the vertebrate hosts present in the study area, and the possibility of mixed blood meals from multiple vertebrate species.

### *Aedes* spp.

*Aedes cinereus* and *Ae. canadensis* fed upon white-tailed deer, albeit the samples sizes were small. Our findings are in agreement with the results of other studies indicating that these mosquitoes demonstrate a propensity for feeding upon large mammals [22, 32–34]. While abundant in Connecticut, the rather few specimens collected in this study was likely due to inefficiency of resting boxes in collecting these species in comparison to CO_2_-baited CDC light traps [35]. Earlier studies have identified *Ae. canadensis* as a potential “bridge vector” of EEEV in epidemic/epizootic transmission based on abundance, relatively frequent virus isolations from field-collected mosquitoes, and vector competence [5, 19, 20, 36]. *Aedes canadensis* has been considered to feed heavily upon turtles. In an earlier study, this mosquito was a dominant species collected from turtles encountered in the wild and from those exposed to mosquitoes, even though several other mosquito species were also numerous at the study sites [37]. *Aedes cinereus* has been reported with relatively high EEEV titers [5], but the contribution of this species to virus transmission is not well defined. Several studies have shown predisposition of *Ae. cinereus* for feeding upon mammalian hosts [22, 32–34], which may limit its role as a “bridge vector”.

As the most abundant mosquito species examined in the present study, *Ae. thibaulti* obtained blood meals from several vertebrate hosts. Earlier blood meal analyses in Connecticut [22] and New Jersey [34] reported *Ae. thibaulti* as an exclusive mammalian biter. However, we identified blood meals from eight mammalian and seven avian host species. The vector competence of this species for EEEV has not been assessed, nor has the virus been isolated from field-collected specimens. Although *Ae. thibaulti* breeds in the same habitats as *Cs. melanura*, its role as a potential “bridge vector” remains unclear.

### *Culex* spp.

Both *Cx. pipiens* and *Cx. restuans* acquired blood meals from avian hosts, supporting their potential contribution to enzootic transmission of EEEV. Close interactions of *Cx. pipiens* and *Cx. restuans* with avian hosts, particularly the gray catbird, red-eyed vireo *Vireo olivaceus* (L.), tufted titmouse, and wood thrush have been reported [38, 39]. These birds are considered important reservoir and amplifying hosts for EEEV throughout the region (e.g., New Jersey, New York, Connecticut and Massachusetts) [10, 11, 16, 40]. Although the role of *Cx. pipiens* and *Cx. restuans* in West Nile virus transmission has been well established [27, 41, 42], their role in EEEV transmission remains to be defined. It is notable that in an earlier study, six isolations of EEEV were obtained from *Cx. pipiens* during an epizootic outbreak in this same region of southeastern Connecticut in 1996 [43], reaffirming the apparent ability of this species to support virus replication.

*Culex territans* has been shown to feed primarily on amphibian and reptilian hosts [30, 33, 44–46], but recent evidence demonstrates that this species feeds on avian and mammalian hosts as well [22, 34, 45, 46]. In our investigation, *Cx. territans* obtained blood meals from avian and amphibian hosts, as well as two mixed blood meals from avian and mammalian species. Several competent or moderately competent bird species, including tufted titmouse and wood thrush, were identified as hosts. Studies indicate that *Cx. territans* may readily feed on several vertebrate classes [34, 45, 46]. Considering recent reports of the involvement of reptilian species in the amplification of EEEV and the blood feeding potential of *Cx. territans* on these vertebrate hosts [47, 48], it is conceivable this mosquito species could transmit the virus among several host classes. EEEV has been isolated from field-collected *Cx. territans* in the northeastern United States [36, 49, 50]; however, the vector competence of this species is not known.

### *Culiseta* spp.

In accordance with the findings of other studies [15, 32, 33, 51], *Cs. morsitans* exclusively acquired blood meals from avian hosts in the present study. Our results, in conjunction with other lines of evidence including abundance, presumed vector competence, and frequent infection in field-collected mosquitoes [2, 12, 22, 51], including this region in Connecticut [43], further establishes the role of this species in enzootic transmission of EEEV among wild birds. Wood thrush served as the most frequent host for *Cs. morsitans*, comprising 63.0 % of the total blood meals. This finding provides further evidence that regional populations of *Cs. morsitans* readily blood feed on wood thrush, consistent with the results of blood meal analysis conducted at an EEEV focus in NY, where 30.9 % (*n* = 42) of blood meals were identified from this species [12]. Using an empirically-informed mathematical model, wood thrush and a few other avian species were identified as potential superspreaders of EEEV at the same virus foci in Connecticut [16]. Notably, the prevalence of wood thrush-derived blood meals from *Cs. morsitans* was greatest during the month of August (28 of 34), similar to that of *Cs. melanura* on wood thrush in the latter study [16]. Other avian species including American robin, gray catbird and tufted titmouse, that served as hosts for *Cs. morsitans*, have been identified as competent or moderately competent amplification hosts for EEEV in the northeastern United States [10, 11, 40].

### *Coquillettidia* spp.

*Coquillettidia perturbans* has been considered as a “bridge vector” of EEEV to humans and equines in the northeastern United States based on its local abundance, vector competence, frequent infection in field collected mosquitoes (including those collected from this region of southeastern Connecticut), and established opportunistic biting [2, 4, 18–20, 22, 32–34, 36, 43, 52–54]. Our identification of EEEV-competent avian species including American robin, black-capped chickadee, and tufted titmouse, as well as white-tailed deer, as hosts for *Cq. perturbans* in the present study support the view that this species could facilitate epidemic/epizootic transmission of the virus.

### *Anopheles* spp.

*Anopheles punctipennis* and *An. quadrimaculatus* obtained the majority of blood meals from mammalian hosts, consistent with the findings of earlier studies [15, 22, 33, 34, 38, 55, 56]. White-tailed deer was the most frequent host for both species, comprising 95.0 and 73.9 % of blood meals for *An. punctipennis* and *An. quadrimaculatus*, respectively. Horse-derived blood meals were also identified from 2.5 % of *An. punctipennis* and 26.1 % of *An. quadrimaculatus*. EEEV has been isolated from field-collected *An. quadrimaculatus* in the eastern United States [4, 5, 20, 55], and sufficient viremia required for subsequent transmission to mammalian hosts has also been reported [5, 19]. Although reports of avian blood feeding by these mosquitoes have been relatively rare, 16.7 % of *An. punctipennis* and 4.0 % of *An. quadrimaculatus* blood meals in the present study were from avian hosts. *Anopheles quadrimaculatus* has been identified as a moderately competent vector of EEEV [19] and is abundant in virus foci, seeking hosts from mid-summer to early fall [20, 35, 55], thus it is plausible that this species serves as an epidemic/epizootic vector of the virus. The vector competence of *An. punctipennis* for EEEV; however, has not been well established.

White-tailed deer served as the most frequent mammalian host for mosquitoes in the present study. Although deer have been considered as dead-end hosts for EEEV [57], the use of these ruminant mammals as indicators of EEEV activity has recently been proposed [58, 59]. Serologic evidence suggests that deer are exposed to EEEV over relatively large geographic regions in Maine, Vermont, and Georgia, in areas not always associated with virus foci [58–60]. Moreover, EEEV has been reported to cause mortality or neurological impairment in deer populations in Georgia and Michigan, and the virus has been isolated from the brain tissue of these mammals [60, 61]. Fatalities caused by EEEV infection in white-tailed deer have also been reported from New York, where infections of a deer and a horse occurred within two weeks of one another in the same town [9]. Nonetheless, the exposure of deer to EEEV does not indicate whether these animals maintain viremia sufficient to infect mosquitoes. The extent of the contribution of a host with any virus titer to transmission has yet to be defined; however, it is conceivable that low viremia could also lend some support to arbovirus transmission. Probabilistic models may be useful in explaining the transmission of an arbovirus from a “dead-end” host, such as white-tailed deer, to an uninfected mosquito [62].

## Conclusions

Our findings indicate that wood thrush, tufted titmouse, and a few other avian species serve as hosts for mosquitoes and likely contribute to amplification of EEEV. Our study supports the role of *Cs. morsitans* in enzootic transmission of EEEV among avian species. *Culex territans* will seek blood from multiple vertebrate classes, suggesting that this species may contribute to epizootic transmission of the virus. Our findings support roles for *Cq. perturbans* and *An. quadrimaculatus* as epidemic/epizootic vectors to humans, horses, and white-tailed deer. Despite its abundance, the potential of *Ae. thibaulti* to serve as a “bridge vector” for EEEV remains unclear in the absence of any definitive knowledge on its competency for the virus. The contribution of white-tailed deer to the dynamics of EEEV transmission is not fully understood, but findings indicate repeated exposure due to frequent blood feeding by vector competent mosquito species.
